# Adaptive mechanisms of developing social connectedness in virtual reality environments

**DOI:** 10.1080/24735132.2025.2506255

**Published:** 2025-06-27

**Authors:** Izabela Derda, Micky Wouter, Tim Smit

**Affiliations:** Media and Communication, Erasmus School of History, Culture and Communication, Erasmus University Rotterdam, Rotterdam, Netherlands

**Keywords:** Social connectedness, social presence, immersion, virtual reality, social virtual reality, social space

## Abstract

Loneliness and social isolation, only exacerbated by the COVID-19 pandemic, are pressing societal challenges. Social Virtual Reality (SVR), as a form of social technology, can be seen as one of the alternatives for those unable to engage in traditional in-person interactions. This paper explores the dynamics of the development of social connectedness in SVR environments, highlighting the roles of embodiment, gestures, and non-verbal cues. The study reveals that the social connectedness in SVR unfolds through three formative phases and that the transition of social spaces from physical to virtual requires active participant engagement in (re-)negotiating and (re-)defining these spaces. The findings underscore the implications for designing virtual interaction spaces to enhance the potential for social connectedness development.

## Introduction

The prevalence of psychosocial problems related to social isolation has been a growing issue for well-being and health and has become a significant societal problem (Koller et al. 2006). The detrimental effects of loneliness and social isolation on overall well-being have been extensively studied (for example, Hawkley et al. 2010, Veldmeijer et al. 2020), highlighting that a considerable percentage of individuals, including approximately 80% of those below the age of 18 and 40% of adults aged 65 years and above, experience occasional episodes of loneliness rather than objective social isolation (Berguno et al. 2004; Pinquart and Sorensen 2001). Still, the implications of these emotional states surpass mere mental distress, as research has established a strong association between feelings of loneliness and heightened risks of morbidity and mortality (Penninx et al. 1997; Sugisawa, Liang, and Liu 1994; Thurston and Kubzansky 2009).

Addressing the psychosocial issues arising from social isolation has gained paramount importance in public health discussions, particularly in the wake of the COVID-19 pandemic. The pandemic has shed a spotlight on the problem of loneliness, elevating it to a critical public health concern and policy priority. Recognizing its significance, the United Nations’ Demographic Change and Healthy Ageing Unit identified loneliness as one of the primary areas of focus during the Decade of Healthy Ageing (2021–2030) [World Health Organization (WHO), 2022].

Interventions targeting loneliness often employ mediated environments to enhance the sense of connectedness. Studies focusing on ‘hidden youth’, who tend to isolate themselves within their homes or bedrooms for extended periods, have highlighted the crucial role of digital technology, particularly online gaming communities, in facilitating their social interactions (Wong 2020). Video calls, on the other hand, have been found to emulate offline social gatherings for geographically dispersed families by allowing multiple family members from both ends of the call to actively participate (Ames et al. 2010; Nguyen et al. 2022). Similarly, Social Virtual Reality (SVR) aims to create a compelling illusion of an unmediated experience, thereby enhancing users’ sense of presence (Lombard and Ditton 2006). Virtual Reality (VR) has also demonstrated the potential to improve mental health outcomes and address conditions like anxiety and depression (Burdea and Coiffet 2003; Riva et al. 2016) as the immersive nature of VR allows individuals to detach themselves from their physical surroundings and immerse in a virtual world, offering a therapeutic escape (Riva et al. 2016). As a result, digital methods, including SVR and VR, assume a crucial role in communication when in-person interactions are impossible, impractical, or less preferable (Baym 2015).

Prior studies have examined diverse facets of employing technologies and virtual reality to amplify social experiences, foster connectedness, and enhance enjoyment. For instance, investigations have explored the significance of digital communication tools in upholding social connections during times of physical separation, emphasizing their capacity to alleviate feelings of loneliness and isolation (Nguyen et al. 2022). Additionally, studies have delved into the role of social presence and interactivity in cultivating empathy within immersive 360-degree videos (Pimentel et al. 2021) or how virtual simulators can be utilized to stimulate perceived enjoyment, value, and behavioural intention (Lee, Chung, and Lee 2013). While recent studies have shown the effectiveness of virtual reality in improving mental health and enhancing connectedness (for example: Riva and Serino 2020; Pallavicini et al. 2016; Gorini et al. 2010; Rus-Calafell et al. 2018; Maples-Keller et al. 2017), the exploration of specific dynamics of interactions leading to experienced social presence and social connectedness, and their implications for the design of virtual interaction spaces is limited. Therefore, this study aims to investigate how social connectedness is developed in social virtual reality environments. As such, the research goes beyond the conventional focus on ‘usability’ and delves into how meaningful connections are forged through mediated interactions.

## Theoretical framework

In today’s increasingly interconnected world, digital communication has become a vital means of connecting individuals and fostering social relationships, while various forms of digital communication play distinct roles in facilitating social connectedness (Nguyen et al. 2022). This observation aligns with the principles of the theory of social presence (Short, Williams, and Christie 1976), which suggests that different media can have varying effects on social connectedness processes (Oh, Bailenson, and Welch 2018, Nguyen et al. 2022). However, in the early studies, social presence was predominantly explored from a technological determinism standpoint, focusing solely on the communication capabilities of the medium (Short, Williams, and Christie 1976). As technology advanced and the internet gained prominence, it became apparent that social presence and connectedness should be examined not only through a technological determinism lens but also by considering the influence of an individual’s social circumstances (Kreijns, Xu, and Weidlich 2022). Hence, by expanding the perspective on social presence, a more comprehensive understanding of how digital communication influences social connectedness can be achieved.

### Social connectedness and social space

Social connectedness refers to ‘the momentary affective experience of belonging to a social relationship or network’ (Van Bel, IJsselsteijn, and de Kort 2008). Van Bel, IJsselsteijn, and de Kort (2008, van Bel et al. 2009) introduced the term referring to belongingness and relatedness when using communication systems, that address experiences along five dimensions, namely: (1) knowing each other’s experiences, understood as an ability to understand the thoughts and feelings of others in the social network, (2) contact quantity^1^, indicating dis/satisfaction with the size of one’s social network, (3) contact quality, being a level of dis/satisfaction with the depth and understanding in social interactions, (4), relationship salience, meaning perceived importance and feeling connected with others in the social network, and (5) shared understanding, describe as a feeling of common interests, ideas, and being on the same ‘wavelength’ with others.

Bel et al.’s (2008) dimensions of social connectedness can be connected to the concept of social space as proposed by Kreijns, Xu, and Weidlich (2022). Social space refers to the interpersonal and emotional connections among group members within a computer-generated environment, encompassing both spatial and cultural aspects. It describes the network of interpersonal relationships embedded in group structures, including norms, values, rules, roles, beliefs, and ideals, and, therefore, indicating that a healthy social space is characterized by a strong sense of community, a positive group environment, mutual trust, social identity, and group cohesiveness. Thus, social space can be seen as a group characteristic (Kreijns, Xu, and Weidlich 2022).

To explore interpersonal interactions and the process of development of participants’ sense of connectedness in SVREs, it is important to examine participants’ characteristics and positions within the social space, as they can have a direct impact on the nature of interactions within the virtual environment (Kreijns, Xu, and Weidlich 2022; Van Bel, IJsselsteijn, and de Kort 2008). Therefore, the theoretical framework for this project is based on the conceptual frameworks of social connectedness proposed by Van Bel, IJsselsteijn, and de Kort (2008) and social space by Kreijns, Xu, and Weidlich (2022).

### Social virtual reality environments

Social virtual reality (SVR) is considered a form of communication allowing people to interact with each other in a virtual environment. In SVR, avatars are used as virtual representations of individuals and enable users to communicate and interact with others using gestures, movements, and expressions. Through this embodied interaction, experience participants can convey messages using both verbal and non-verbal cues. Avatars, therefore, act as proxies for communicators to engage with one another and express themselves in the virtual space (Manninen and Kujanpaa 2007). The use of animated representations in SVR environments (SVRE) can influence the perception of social interactions, thus, precise positional tracking of the body and objects is crucial in maintaining the illusion of three-dimensional space and enhancing the realism of the VR experience (Desai et al. 2014), and as such, potentially affecting the feelings of presence and immersion.

In addition to audiovisual content, social VR allows for the incorporation of sensory information through haptic technology, which simulates the sense of touch. Haptic technologies involve solutions that detect and replicate contact, providing real-time interaction feedback through haptic devices (Burdea 1999), like gloves, vests, suits, or masks. The inclusion of haptic cues as a means of non-verbal communication in social VR has the potential to positively influence social interactions, enrich storytelling, and enhance narrative engagement (Fermoselle et al. 2020; Rasool, Molka-Danielsen, and Smith 2021).

In the context of SVRE, the concepts of presence and immersion are critical for understanding the impact of technology. Drawing on the understanding of social presence (Kreijns, Xu, and Weidlich 2022) as the perception of the ‘realness’ of other participants, it can be understood as a subjective experience of being present in the virtual environment (Walther and Parks 2002; Witmer and Singer 1998). On the other hand, immersion relates to the technology’s ability to create a convincing illusion of being somewhere else, as measured by the sensorial information provided to the user (Cummings and Bailenson 2016; Fermoselle et al. 2020; Skalski and Whitbred 2010; Slater and Wilbur 1997). Immersion then focuses on the objective attributes of the medium allowing for the feeling of creating illusion (such as audiovisual or haptic feedback quality), while presence encompasses the user’s engagement and subjective experience within the virtual world.

As social VR is still an evolving communication tool, it is important to consider its technological attributes and affordances when examining social interaction within this environment. By understanding the dynamics of interactions, the impact of embodiment, gestures, and non-verbal cues, as well as the interplay between presence, immersion, and social space, the potential of social VR for fostering social connectedness can be explored.

## Methodology

This research aimed to investigate individual experiences of shared interactions and the potential for the development of social connectedness in SVREs, through the perspective of the concepts of social presence and social space, focusing on embodiment, gestures, and non-verbal cues. To achieve this, a qualitative explorative approach was employed, as it is well-suited for identifying respondents’ views, expectations, and experiences (Boeije 2014).

As this study focused on the *process* of developing social connectedness in virtual spaces, the paradigm model proposed by Corbin and Strauss (2008) was adapted to structure the research. This approach enabled the identification of conditions (e.g. technological affordances, participant relationships), actions/interactions (e.g. use of gestures, renegotiation of social space), and consequences (e.g. levels of connectedness and engagement) observed in participant experiences. As such, the study followed an inductive approach using grounded theory (Glaser and Strauss 1967), supported by thematic analysis as described by Braun and Clarke (2013). While the theoretical framework informed the research design and the development of the interview guide, the subsequent analysis was data-driven. Patterns and themes emerged organically from the data and were then compared with existing theories to generate new insights.

Throughout the study, data collection and analysis occurred in continuous interplay. The data was collected in two waves, and the insights from earlier observations and interviews informed subsequent data collection, ensuring that emergent themes were explored further. For instance, during the analysis of the second dyad, it was observed that a mirror served as a helpful tool for participants to embody themselves in the virtual environment. This observation was further investigated with subsequent dyads to determine whether it was a single occurrence or part of a broader pattern. Such observations were also incorporated into interview questions to better understand participants’ motivations behind their actions.

The analysis process involved three iterative stages: open coding, axial coding, and selective coding (Braun and Clarke 2013). Both observation and interview data were considered during this process. Open coding entailed a detailed examination of the data—line by line for interviews and second by second for observations—to identify initial codes that captured participants’ behaviours and experiences. Axial coding then grouped these codes into categories based on relationships and shared themes. Finally, selective coding integrated these categories into overarching themes, which contributed to the proposed model of social connectedness development in SVREs. This iterative process allowed for the refinement of categories and provided a deeper understanding of how social connectedness evolves in SVREs.

### Research environment

The research utilized two different SVREs for data collection. The rationale behind using these two different environments was to create a comprehensive understanding of user experiences across varied social VR scenarios. In this case – one offering casual social interactions and more freedom to users on how to interact within the space and the second more structured, to challenge users with collaborative tasks within VR environments.

The first environment was VRchat, an existing social virtual reality platform designed to resemble a real-life social setting (VRChat, n.d.). In VRchat, participants were placed in a virtual café environment, complete with indoor and outdoor areas featuring various elements such as tables, chairs, a bar, a pool table, a couch, a television, and a mirror. The participants were instructed to engage in simple tasks, including interacting with the environment, picking up objects, opening and closing curtains, talking to each other, exchanging items, looking at the mirror, and playing games using a pool table, and a table with cups on it into which participants were asked to throw a ball in.

**Figure 1.**
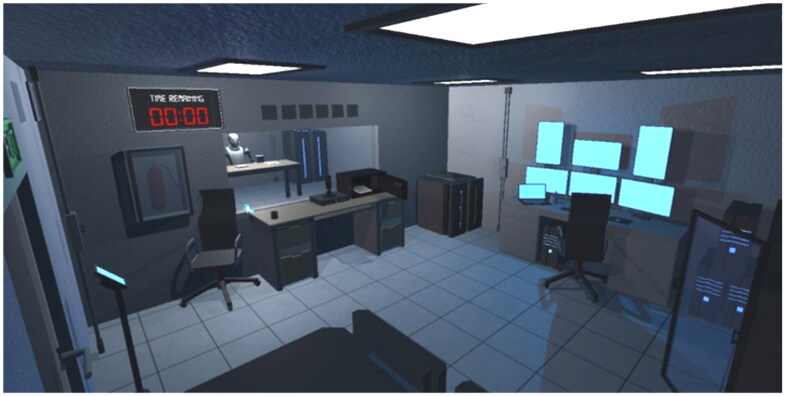
Kyle’s escape experience (player 1 view).

**Figure 2.**
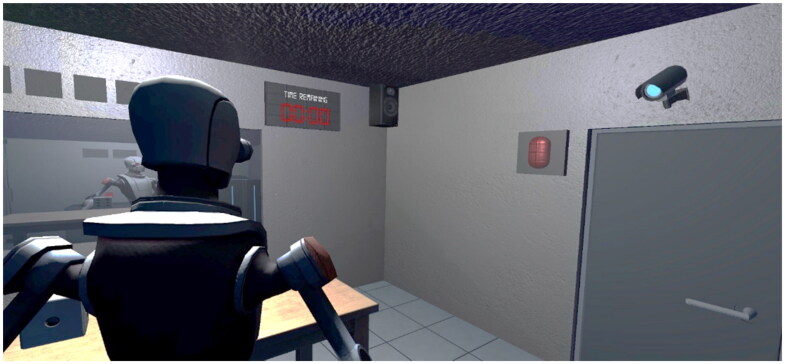
Kyle’s escape experience (player 2 view).

The second environment used in the research was ‘Kyle’s Escape’, a Virtual Reality escape room designed for the research and created in Unity (Figures 1 and 2). The game consisted of two phases both focusing on collaboration and the objective of escaping from the room. The first phase aimed to introduce players to cooperative play in a virtual reality environment. Players began in separate rooms with a one-way mirror, limiting their visibility of each other, and providing them with an environment restricting the (direct) use of haptics in interpersonal communication. In the second level, both players worked together to overcome obstacles, such as locked doors and puzzles, within a time limit.

The prototyped environment facilitated social interactions and challenge-based experiences and examined how users can create social presence through virtual interaction, by incorporating hand/body tracking technology to express natural body language gestures. In both environments, specific hardware was used to deliver the experience and provide tactile feedback during interactions with the virtual elements: 2 Oculus Quest 2 headsets, 2 Manus Prime X haptic gloves, 2 bHaptics TactSuit X16 vests (16 feedback points), 2 sets of bHaptics Tactosy for arms (12 feedback points per set). Both Manus and the bHaptics Designer tool were utilized to refine haptic feedback for VRChat and design relevant feedback for Kyle’s Escape game. The setup underwent trial research involving three dyads (not included in further research) to assess feasibility and attain the most suitable and authentic user-perceived feedback achievable within the designated framework and given hardware setup.

To enable social interaction, the experiment was conducted with dyads, consisting of two communicators. The total sample size consisted of 16 participants, forming 8 couples, of which six couples were categorized as having strong relational ties (e.g. friends, loved ones, family members) and one with weak ties (e.g. acquaintances, strangers). However, it is important to clarify that the primary objective of this study was not to compare and contrast the dynamics of different types of social ties in virtual reality environments. Instead, the focus was to understand the broader dynamics of how participants negotiated social space and (if they) developed social connectedness within such environments.

Each couple engaged in both experiences, which were presented to them as unrelated. Couples were not tasked with transferring learnings from one experience to another, although for those with limited VR experience, this occurred in a natural way. The couples were briefed on the experiences, provided with instructions, and given time to familiarize themselves with the use of the provided hardware and navigating in a virtual environment. Dyads were separated during VR experiences to prevent auditory or physical interaction within the physical space. They also were not permitted to discuss coping strategies before the first experience or debrief each other before the second, allowing exploration of natural coping mechanisms and navigation strategies in social space within the given environments.

Their interactions were recorded, and detailed notes were taken. Following the experiences, participants were individually interviewed about their experiences. During the interviews perceived social connectedness was explored through the combined, shortened, and adapted for qualitative explorations of Lee and Robbins (1995), Van Bel, IJsselsteijn, and de Kort (2008), Kreijns, Xu, and Weidlich (2020; 2022).

The research, which involved non-sensitive groups, strictly adhered to the Netherlands Code of Conduct for Research Integrity. All participants provided informed consent and were afforded both anonymity and the opportunity to withdraw from the study at any point.

## Results

**Figure 3.**
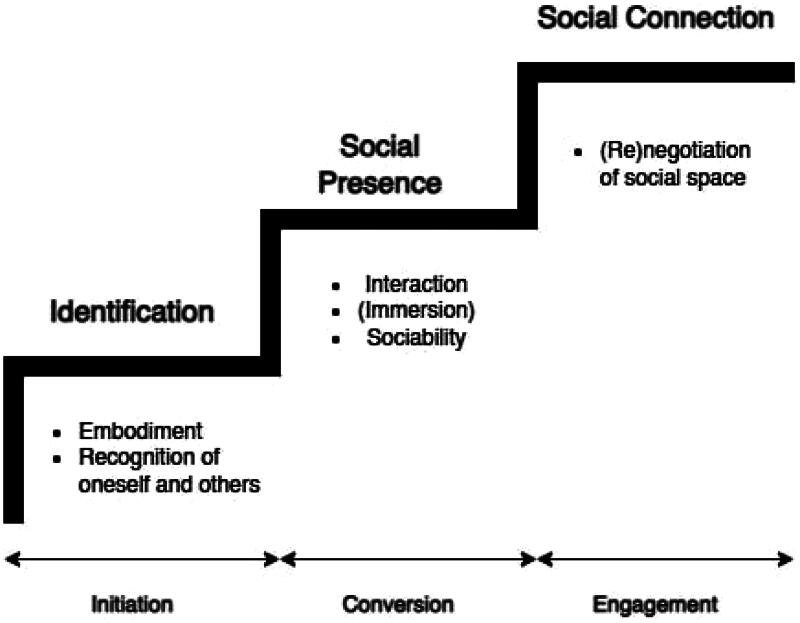
Model of social connectedness development in SVREs.

The analysis of participant observations and interview data revealed that the development of social connectedness unfolds through three distinct phases: (1) initiation, (2) conversion, and (3) engagement. These phases are depicted in Figure 3, which presents the Model of Social Connectedness Development in SVREs.

### Initiation

#### Identification

The initiation phase can be seen as a critical stage in the development of social connectedness within SVREs. During this phase, participants engage in the process of (self-)identification, which can be viewed as a precondition for experiencing a sense of realness and presence in the virtual environments as previous research has emphasized the significance of identification in shaping the experience of social presence (Walther 1993). Our findings align with this notion, revealing that the animated representation of avatars hindered the participants’ ability to establish a strong sense of identification. The visual portrayal of avatars, lacking human-like features or providing minimal personalized characteristics, posed a barrier to perceiving the self and another person as real. However, it was discovered that gestures and body language (through the intermediate introduction of a mirror in the virtual environment) proved instrumental in grounding participants’ sense of reality and bridging the gap in identification. Through the incorporation of interactive hand/body tracking features, participants were able to effectively convey gestures and movements. As participants stood in front of the virtual mirror, they engaged in the ability to mimic dance moves and lively gestures, witnessing their avatars mirror their every action with high accuracy, forging a tangible connection between their physical selves and virtual counterparts. This allowed them to directly see and interact with their virtual representations, leading to a heightened sense of embodiment and bridging the gap between their physical selves and virtual identities.

Body tracking together with spatial and gestural behaviour was also acknowledged as contributing to the feeling of identification. Participants addressed the fact that they recognized the way the other participant was moving through the virtual reality environment, which contributed to recognition. They have also reported that the realistic portrayal of gestures enabled them to recognize familiar traits and personalities, fostering a deeper level of connection and grounding them in the environment:

They also reported that the realistic portrayal of gestures enabled them to recognize familiar traits and personalities, fostering a deeper level of connection and grounding them in the environment. This perceived realism also played a crucial role in helping them identify and recognize specific characteristics of co-participants. Through the positional tracking offered by immersive VR, the height difference between real-life participants was translated well into the avatars. Although the animated characters did not resemble a representation of human beings, nor was a unique representation of the participants themselves, the height difference ensured that there was some recognition. The fact that the participants in most cases knew each other in real life contributed to assigning characteristics such as the height of an avatar to the resemblance with the other person. Participants also addressed the fact that they recognized the way the other participant was moving through the virtual reality environment:

I really saw myself and Fleur, also because she was smaller. I really liked that, because that just made it feel more like her and, for example, when she was at the pool table, that [it felt like] she really stood in front of me…I was moving backward [to make her space] while she also didn’t quite touch me, but that made it feel like she was very close to me. (Interviewee 7)

Moreover, participants have reported that the realistic portrayal of gestures enabled them to recognize familiar traits and personalities of their co-participants they knew from real life, fostering a deeper level of connection and grounding them in the environment:

Yes, of course, I recognize her… and she’s a little clumsy [in real life]. So, when I see her [in the virtual environment], I see her jumping around clumsily and struggling to get off a bench, just like she always does. (Interviewee 3)

Other participants also mentioned the gestural movement of the other as contributing to recognizing someone’s real-life characteristics. The use of gestures and non-verbal cues proved pivotal in enhancing the initiation phase of social interactions. Replicating gestures and providing participants with a sense of physically moving their hands and bodies in a manner consistent with the physical world contributed to the experience of identification. Participants expressed familiarity in recognizing the unique physical traits and behaviours of their co-participants, which further solidified their sense of presence and connection. As Interviewee 9 expressed:

I remember saying at one point: I can just see from the way she moves that it’s her. The way she plays, just how she stands by that table and tries to grab things and move. And when she’s playing a game, then all that body goes with it. So, then she stands there and then she jumps and then you see the whole avatar going in all directions when she throws a ball, so that’s funny, I recognized that immediately. That you immediately recognize someone’s personality in the real world.

### Conversion

#### Social presence – positional tracking, immersion, and sociability

As Slater (2018) suggests, presence in virtual environments can be understood as a subjective psychological response to a VR system. In this context, the perception of ‘realness’ illustrates the malleability of presence within virtual environments and highlights individuals’ capacity to find (alternative) ways of establishing identification and connecting with others. Therefore, the *conversion phase* builds upon identification and serves as a gateway to developing social connectedness.

The participants reported experiencing presence across four dimensions: resemblance with the real world, interaction with the virtual world, interaction with other participants, and embodiment illusion. The four dimensions can be linked to *immersion* and *sociability* as factors contributing to the feeling of presence.

The realistic resemblance of the virtual environment and its objects to the physical world, coupled with the ability to engage in physical interactions such as picking up, playing with, or throwing objects using gloves-covered hands instead of traditional VR controllers, significantly contributed to the perception of the environment as ‘real’. This aligns with the concept of immersion understood as the extent to which the sensory stimuli provided by the virtual environment are perceived as being real and the extent to which the user is able to interact with and navigate within the virtual world (Cummings and Bailenson 2016; Oh, Bailenson, and Welch 2018; Skalski and Tamborini 2007; Slater and Wilbur 1997) and allows to say that the environment facilitated the development of immersive experiences. However, it was noted that the technology used in the study failed to generate full immersion. Participants mentioned various technological constraints that hindered immersion, such as haptic feedback, visual information, and animated representations. What is interesting in the context of this study, they pointed to haptic technology specifically as one of the direct barriers to immersion development. Participants expressed that on the one hand, the ability to use their hands and body in a real-life manner for interaction and self-expression helped them immerse in the experience (as opposed to the use of traditional VR controllers). However, on the other hand, the bulkiness of the hardware was making them constantly conscious of the presence of the technology and was pulling them out of immersion. As haptic technology (that was used for this research) falls short of replicating authentic physical touch (using vibrations) or feel of the objects (failing to mimic the actual weight of objects or strength of hold or touch) provided by the gloves, vests or suits are not enough to mimic tactical sensations in a believable manner.

As in the case of identification, limitations of the technology were compensated by the participants’ way of interacting with themselves and the provided environments. Sociability, understood as the capacity of immersive VR to allow for the expression of social presence and the emergence of social space (Kreijns, Xu, and Weidlich 2022), was positively perceived by participants. Social affordances offered by the virtual environment, such as games they could play together and opportunities for communicating with each other through verbal and non-verbal, facilitated social interaction and, in effect, ‘grounded’ them more in the experience. The participants emphasized the realness of the experiences they shared with others and the sense of doing something together.

There is a real experience that you share with someone. So, it actually feels very real, because we did it together, communicated with each other, and played a game. Therefore, it was definitely that we did something real with the two of us. (Interviewee 4)

The recognition of social cues, active social interaction, and the establishment of emotional connections among participants played a significant role in fostering the development of presence, surpassing the limitations posed by animated representations and technological constraints. Consequently, sociability emerged as a crucial factor in the advancement of social connectedness.

### Engagement

#### Development of shared social space

In the realm of social virtual environments, the development of social space holds a significant role in fostering interpersonal connections and facilitating a sense of connection among participants. During the *engagement phase*, it becomes particularly relevant, as it becomes evident that certain dynamics, structures, rules, and connections between the participants (having both strong and weak ties) were established and influenced the development of social connectedness. For example, the social interaction in the SVRE was ruled by the type of ties between the participants and affected by gender. This was exemplified by Interviewee 5 (a participant paired up with a person he did not know before, therefore, belonging to a couple representing weak ties), who expressed discomfort in giving his (female) co-participant a hug within the virtual environment. Interviewee 5, being a man, hesitated to initiate digital physical contact with Interviewee 6, a woman, even in the situation when it could be seen as a relevant reaction to the happenings, as he found it peculiar to approach her avatar and touch her representation in the virtual environment as they ‘didn’t know each other that well’ (Interviewee 5).

It became apparent that the development of social space and, consequently, social connectedness was deeply influenced by the processes of identification and presence development in the initiation and conversion phases. As one participant expressed, ‘I felt someone’s presence, but not necessarily him specifically [as I know him in real life]’ Interviewee 6. This quote suggests that the participant did not perceive her co-participant as ‘real’, and as a result, she did not experience the same level of comfort as in a physical environment. Verbal and non-verbal cues only partially compensated for this disparity.

While the dyads with strong ties found it relatively easier to establish identification and quickly attain a state of social presence in the virtual environment, this did not automatically allow for replicating the same social space they shared in real life with their co-participants. Consequently, some groups with strong ties necessitated a re-evaluation of their existing social space and the establishment of new frameworks for their relationship. The groups with weak ties had to develop a social space while being in SVRE.

Social connectedness was successfully established among all dyads participating in the study. However, it is important to note that the progression through the phases of initiation, conversion, and engagement took varying forms and durations for different participants. While all participants and dyads demonstrated a combination of behaviours outlined in Table 1, the process was not always linear, nor did it take the same shape for everyone. For instance, during the initiation phase, the process of identification was achieved by all participants, but the pathways differed. Individuals with prior familiarity with VR technology reached self-identification quickly (14–48 seconds), often through simple body movements. In contrast, participants less experienced with VR required more time and actions (up to 8 minutes), such as experimenting with gestures or engaging with their avatars, to familiarize themselves with the technological affordances and embody themselves in the experience.

**Table 1.** The role of embodiment, gestures, and non-verbal cues in the development of social connectedness in SVR.

Phase	Embodiment	Gestures	Non-verbal cues
Initiation	Establishing identification through avatar representation Bridging the gap between physical and virtual selves	Mimicking (real-life-like) gestures and movements through hand/body tracking Facilitating (non-verbal) communication and expression	Recognition of familiar traits and personalities Enhancing the sense of presence and connection
Conversion	Grounding participants in the virtual environment Realistic interactions contribute to a sense of reality	Enhancing immersion and sense of presence Supporting social engagement	Recognizing social cues and emotional engagement Compensating for disparities in perceived realism
Engagement	Influencing comfort levels and interactions Navigating social space and establishing connections	Negotiating social dynamics and boundaries Establishing boundaries and navigating social dynamics	Shaping perceptions of social presence and connection Facilitating negotiation and adaptation for meaningful connections

What was crucial for shaping the Model of Social Connectedness Development in SVREs (Figure 3) were turning or decisive moments that clearly marked participants’ progression to another phase. These moments were observed across all dyads and participants, even if the paths leading to them varied. In some cases, the transition was expressed verbally. For example, one participant explicitly stated, ‘Ok, I get it now, should we play something?’—marking the closure of actions related to self- and other-identification and the progression to interaction and sociability. In other instances, the change was represented through behaviour, such as progression from one activity to another type of activity, and later reflected upon during the interview phase. One participant commented, ‘I said that because I felt like we are fine with that [what was happening in the initiation phase] and was ready to do things [activities available in the SVRE]’ (Interviewee 6).

While some participants revisited earlier phases during their interactions, these moments were seamlessly integrated into their overall progression. For instance, one dyad that had entered the engagement phase chose to change their activity, which prompted them to revisit the identification phase to reconfirm the boundary conditions of their interaction. Once the identification was re-established, they resumed the engagement phase without issue. Instances, where participants revisited earlier phases, were included in the results only if they introduced new insights or conditions relevant to the development of social connectedness.

It is worth noting that approximately half of the dyads encountered the need to *(re)negotiate their social space* (for others this process … This indicates that while social connectedness was achieved, the nature of the social space had to be reconsidered and adjusted for a significant portion of the participants. This highlights the dynamic and evolving nature of social interactions in immersive virtual environments and underscores the importance of ongoing negotiation and adaptation to foster a sense of belonging and connections.

## Discussion and conclusions

The findings of this research indicate that social connectedness within SVREs is developed across three formative phases: initiation, conversion, and engagement. The adaptive nature of individuals in seeking non-verbal cues, and their ability to (re-)negotiate and (re-)establish social space, enhances the potential for the development of social connectedness within SVREs. The results also indicate that in each phase, embodiment, gestures, and non-verbal cues played a role in participants’ feelings of presence, realness, and immersion, thereby contributing to the development of the experience of social connectedness (Table 1).

During the *initiation phase*, the incorporation of interactive hand/body tracking features allowed participants to effectively convey gestures and movements, and, therefore, (self-)identify and connect with others. As much as a representation of others as avatars was identified as the most significant difference from face-to-face communication, the use of avatars did not hinder the development of social connectedness. In line with Walther’s (1993) theory of impression formation and impression management, which suggests that individuals adapt to the constraints of the communication medium, participants of this study demonstrated a high level of adaptability by actively seeking non-verbal cues (for both identification and communication) and engaging in social interactions. Therefore, technology supported participants in developing social presence as they were physically interacting with objects and co-participants. This adaptability highlights the malleability of the perception of ‘realness’ in the virtual realm and the capacity to stretch and adjust it based on available cues.

Linked to that, in the *conversion phase*, technologies played a pivotal role in enhancing participants’ sense of social presence. Through the utilization of social affordances offered within the virtual environment (such as interactive games challenging participants not only intellectually but also physically), participants were able to engage in joint activities, fostering a sense of collaboration and shared experiences. This collective participation, embodiment, and the use of both verbal and non-verbal cues reinforced participants’ perception of the realness of experiences shared with others and the sense of doing something together, thereby being actively present together within the virtual environment. This aligns with the theory proposed by Kreijns, Xu, and Weidlich (2022), which emphasizes the role of medium attributes in social interaction and sociability. As media attributes can include both physical characteristics of the medium and its ability to facilitate the transfer of verbal and non-verbal cues, in the case of immersive SVREs, the social affordances of VR allow for social interaction within the environment (Kreijns et al. 2004; Kreijns, Xu, and Weidlich 2022).

**Figure 4.**
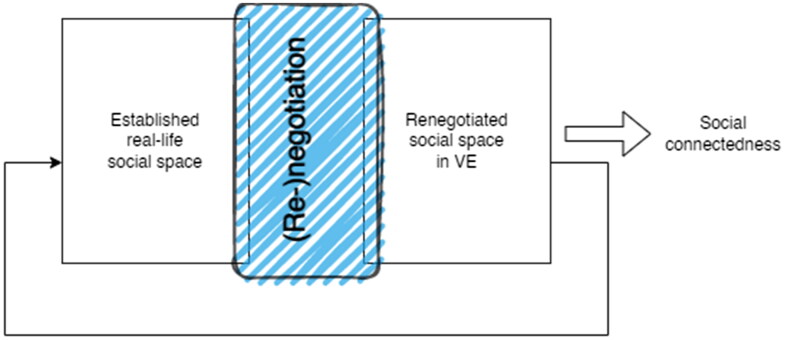
(Re-)negotiation of social space in VE.

In the *engagement phase*, participants, who had already established identification and achieved certain levels of social presence, embarked on the process of *(re)negotiating the social space* within the virtual environment. It was observed that for multiple participants, their real-life social space differed from the one developed in the virtual space. This finding suggests that the transfer of the established social space does not transfer directly nor seamlessly from the physical realm to the virtual. Instead, participants in the virtual environments need to re-evaluate and redefine the social space, including dynamics, structures, rules, and connections between participants (as defined by Kreijns, Xu, and Weidlich 2022) in order to establish a new social space within the virtual environment. Importantly, the social space in real life remains unaffected after disconnection with SVRE, highlighting, what we could call, the reconstructive nature of social space in the virtual realm (Figure 4).

While most dyads successfully renegotiated their social space, there were instances where challenges emerged. For example, in one dyad, a participant expressed discomfort with initiating digital physical contact, such as an avatar-based hug, due to limited familiarity with their partner in real life. This hesitance led to a slower progression through the engagement phase, which, in turn, affected their ability to fully engage in shared activities and effectively slowed down the process, and potentially effected the level of the social connectedness they achieved. However, it is important to note, however, that this research did not aim to evaluate the ‘level’ or ‘strength’ of social connectedness experienced by participants. Instead, the focus was on understanding the dynamic process through which social connectedness develops. Yet, such cases underscore the critical role of trust and familiarity in facilitating seamless renegotiation of social space, highlighting how individual differences and relational contexts can shape the trajectory of the process.

The findings of this research indicate, that the (re)negotiation and reconstruction of social space within the virtual environment are essential for the development of social connectedness. As the transfer of social space from the physical realm to the virtual one is not direct, by establishing (partially) new sets of codes and frames, participants of the SVR experience work together towards creating a shared social space that fosters social connectedness in the virtual realm.

The findings have direct implications for the design practice and emphasize the importance of intentional efforts in shaping and adapting virtual environments, so they can stimulate identification, social presence, and sociability, therefore, facilitating meaningful connections and supporting participants in the development of social connectedness in the virtual settings. More specifically, the findings of the research can be translated into specific design considerations:

- **Environmental transformation**: The transition hiccup underscores the imperative for designers to not merely mimic physical social structures in the virtual sphere. Instead, SVREs must be crafted as dynamic ecosystems that respond adeptly to the virtual milieu, allowing for the organic emergence of new social spaces,
- **Cognizant interface framing**: Given the pronounced role of non-verbal cues in the adaptation process, designers must weave interface elements that are attuned to these dynamics. Crafting intuitive, multimodal interfaces that facilitate the seamless exchange of gestures and expressions can bridge the transition chasm effectively,
- **Facilitation of (self-)identification**: The employment of interactive mechanisms (such as mirrors and/or body tracking features), where participants can discern their virtual reflection through gestures and body movements, can serve as a crucial milestone in enabling participants to recognize themselves and others within the digital realm. Therefore, SVREs should thoughtfully integrate elements that allow for seamless, haptics-based, and intuitive self-recognition,
- **Guided social reframing**: Recognizing the necessity for participants to consciously renegotiate their virtual social framework, designers should integrate features that prompt and support this reconfiguration. Visual cues or interactive modules that invite participants to collaboratively define their virtual social dynamics can bolster this transition,
- **Engagement encouragement**: The transition from physical to virtual amplifies the need for SVREs to transcend mere utilitarian functionality and embrace engagement-driven design. Immersive activities that encourage shared experiences, collaborative problem-solving, and joint explorations within the virtual realm can accelerate the process of social space reconstruction,
- **Enhancement of non-verbal communication**: To leverage the crucial role of non-verbal cues, embodiment, and gestures in developing social connectedness, designers should consider and implement advanced tracking systems and design experiences in a way that would allow for a (realistic) non-verbal self-expression. The avatars should accurately capture and represent users’ gestures and body language, enabling more natural and meaningful interactions. This will improve participants’ self-identification process and help them feel more immersed and connected in the virtual environment,
- **Contextual empathy augmentation**: Informed by the study’s insight into the multi-layered negotiation of social space, designers can infuse SVREs with contextual cues that evoke empathy. Presenting scenarios or stimuli that elicit shared emotional responses can catalyse a more profound and authentic social space transformation.
- **Sociability nurturing**: The transition challenge underscores the significance of sociability as a design cornerstone. Designers must interlace SVREs with affordances for social interactions that mimic real-world dynamics, prompting users to organically define their social space as they interact and bond.

It is important to acknowledge the limitations of our study, such as the small sample size and the specific context of the two tested SVR environments and selected hardware setups. Instead of testing the effectiveness of particular setups in the development of social connectedness, the study focused on a qualitative exploration of the dynamics of social connectedness development in SVRs generally. Furthermore, this study was purely explorative, and no control group was included, nor was a comparative study conducted. As the purpose was to explore the dynamics of the development of social connectedness, this could be the focus of future research. Moreover, future research should aim to explore and expand upon our findings in diverse settings and populations to strengthen the validity and generalisability of the proposed social connectedness development model and reconstructive social space theory. Additionally, investigating the long-term effects of social interactions and exploring the potential of haptic technologies to bridge social disparities in virtual environments are areas worthy of further exploration.

## Note

1 This dimension is irrelevant to this study as it pertains to the satisfaction or dissatisfaction with the quantity and size of one’s social circle, as well as the desire for more friends and a greater number of close relationships. It encompasses feelings of lacking companionship, desiring a larger social network, and perceiving a deficit in social relationships, while this study focuses on the connectedness between two people of either stronger or weaker social ties utilizing a virtual environment as a means for mediated communication.
